# ARA 290, a peptide derived from the tertiary structure of erythropoietin, produces long-term relief of neuropathic pain coupled with suppression of the spinal microglia response

**DOI:** 10.1186/1744-8069-10-13

**Published:** 2014-02-16

**Authors:** Maarten Swartjes, Monique van Velzen, Marieke Niesters, Leon Aarts, Michael Brines, Ann Dunne, Anthony Cerami, Albert Dahan

**Affiliations:** 1 Department of Anesthesiology, Leiden University Medical Center, P5-Q, 2300 RC Leiden, The Netherlands; 2 Araim Pharmaceuticals, Tarrytown, NY 10591, USA

**Keywords:** ARA 290, Spared nerve injury, Allodynia, Dorsal horn, Microglia, Iba-1, Astrocytes, GFAP

## Abstract

**Background:**

Neuropathic pain is a difficult to treat disorder arising from central or peripheral nervous system lesions. The etiology of neuropathic pain consists of several overlapping pathways converging into an exaggerated pain state with symptoms such as allodynia and hyperalgesia. One of these pathways involves activation of spinal cord microglia and astrocytes, which drive and maintain the inflammatory response following the lesion. These cells are a potential target for drugs for neuropathic pain relief. In this current study, we investigated the dose-effect relationship of the tissue protective peptide ARA 290, derived from the tertiary structure of erythropoietin, on allodynia and concurrent spinal cord microglia and astrocytes.

**Results:**

Following a spared nerve injury in rats, vehicle or ARA290 (administered in either one of 4 doses: 3, 10, 30 and 60 μg/kg) was administered on days 1, 3, 6, 8 and 10. ARA290 exerted a dose–response effect by significantly reducing mechanical allodynia up to 20 weeks when compared to vehicle. The reduction of cold allodynia was significant up to 20 weeks for the doses 3, 10, 30 and 60 μg/kg when compared to vehicle. The effect 10 and 30 μg/kg ARA290 and vehicle on the microglia response (iba-1-immunoreactivity, iba-1-IR) and astrocyte reaction (GFAP-immunoreactivity, GFAP-IR) was investigated in animals surviving 2 (group 1) or 20 (group 2) weeks following lesion or sham surgery. In group 1, significant microglia reactivity was observed in the L5 segment of the spinal cord of animals treated with vehicle when compared to sham operated, while animals treated with 10 or 30 μg/kg did not show a increase. In group 2, a more widespread and increased microglia reactivity was observed for animals treated with 0 and 10 μg/kg when compared to sham operated animals, indicated by involvement of more spinal cord segments and higher iba-1-IR. Animals treated with 30 μg/kg did not show increased microglia reactivity. No difference in astrocyte reaction was observed.

**Conclusions:**

The erythropoietin-analogue ARA290 dose-dependently reduced allodynia coupled to suppression of the spinal microglia response, suggestive of a mechanistic link between ARA290-induced suppression of central inflammation and relief of neuropathic pain symptoms.

## Background

Neuropathic pain (NP) is a debilitating condition resulting from lesions of the peripheral or central nervous system with allodynia and hyperalgesia to mechanical or thermal stimuli as main symptoms [1,2]. Treatment of NP is difficult and management of symptoms by pharmacological (opioids, antidepressants or topical agents such as capsaicin) or non-pharmacological (physiotherapy) means is often not adequate. The mechanisms underlying NP are largely driven by peripheral and central inflammation leading to peripheral and central sensitization. Peripherally, macrophages and T-cells are the main contributors to the inflammatory response [3,4]. In the central nervous system astrocytes and microglia play a crucial role in NP states after peripheral nerve injury by showing altered numbers, morphology and activation states [5-11]. There is ample evidence for crosstalk between neurons and glia cells leading to phenomena that underlie allodynia and hyperalgesia [12]. In NP, glia become more abundant and activated as a result of the induced release of proliferative molecules, such as fractalkine (chemokine (C-X3-C motif) ligand 1; CX3CL1) and C-C motif chemoreceptor ligand 2 (CCL2) released by neurons due to increased afferent signaling [13-15], and local release and retrograde transport of TNF-α [16]. These glia cells are involved in driving and maintaining the inflammatory response, especially in the dorsal horn of the spinal cord, by releasing inflammatory mediators, including TNF-α, interleukins 1β and 6 and other signaling molecules for periods that may extend over 2 weeks [17-20]. In addition to the observations in experimental animal studies, a case report of a patient with longstanding complex regional pain syndrome describes increased activation of astrocytes and microglia in the spinal cord after autopsy when compared to patients without a neuropathic pain condition [21]. These observations strongly suggest that astrocytes and microglia serve as potential targets for treatment of neuropathic pain. Indeed, inhibition of activated microglia and astrocytes reduces neuropathic pain symptoms *in vivo*[22-24].

We recently showed that the neuroprotective synthetic 11-amino acid erythropoietin (EPO) derivative ARA 290 produces effective and long-term pain relief following peripheral nerve damage in the rat [25,26]. ARA 290 produces its effects via activation of the β-common-receptor [25-27]. The β-common-receptor in conjunction with the EPO receptor forms a heterocomplex (designated the innate repair receptor, IRR), which becomes locally up-regulated following tissue injury [28,29]. Its activation initiates a local anti-inflammatory response, inhibition of death signal and anti-apoptosis, thereby preventing overt tissue damage. Additionally, activation of the IRR also promotes tissue repair responses, including neurite outgrowth in the nervous system [30]. In humans we recently showed that chronic ARA 290 administration reduced pain symptoms and improved functionality in patients with chronic neuropathic pain related to small fiber neuropathy [31]. Various animal studies have shown the tissue-protective effects of ARA 290, all related in part to its anti-inflammatory effects. For example, ARA 290 improves survival following myocardial infarction, reduces organ dysfunction in hemorrhagic shock and suppresses development of atherosclerosis in hyperlipidemic rabbits [32-34].

In this study, we investigated the dose–response effect of ARA 290 on mechanical and thermal allodynia in an experimental rat model of chronic neuropathic pain (using the spared nerve injury model in which two of the three branches of the sciatic nerve are surgically cut). Next, to better understand its mechanism of action, we assessed whether ARA 290 has an anti-inflammatory effect at the level of the spinal cord by visualizing spinal astrocyte and microglia using immunohistochemistry. We hypothesize that ARA 290 reduces the neuroinflammatory response in chronic neuropathic pain.

## Results

### ARA 290 reduces mechanical and cold allodynia in a dose-dependent manner

#### ***Mechanical allodynia***

**Figure 1:**
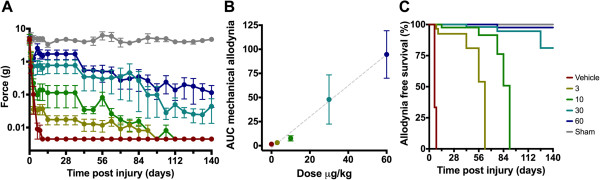
**Effect of ARA 290 on mechanical allodynia. A**. Effect of spared nerve injury and treatment with vehicle or different doses of ARA 290 on mechanical allodynia. Animals were sham-operated (grey) or received spared nerve injury and 5 doses of vehicle (red), 3 μg/kg ARA 290 (yellow), 10 μg/kg ARA 290 (green), 30 μg/kg ARA 290 (marine blue), or 60 μg/kg ARA 290 (dark blue) administered on days 1, 3, 6, 8 and 10 post-surgery. **B**. Correlation of ARA 290 treatment dose and the relief of mechanical allodynia, calculated by the difference in area under the curves (AUC) of the mechanical allodynia response on day 1 vs. day 140. The adjusted R^2^ is 0.56. **C**. Survival analysis showing the mechanical allodynia-free proportion of animals in time either sham-operated, or receiving spared nerve injury and treated with vehicle or different doses of ARA 290. Log-Rank p < 0.001.

Following SNI, vehicle-treated animals progressively developed mechanical allodynia within 10 days with withdrawal responses to the filament exerting the lowest possible force (0.004 ± 0.0 grams). Sham operated animals showed no decline in response threshold. Regardless of treatment, all SNI groups differed significantly from sham operated animals (*p* < 0.001 for all groups). The two-week treatment with ARA 290 produced a lasting relief of tactile allodynia (Figure 1A, treatment effect *p* < 0.001). *Post hoc* analysis revealed significant effects for the 30 and 60 μg/kg groups (30 μg/kg: *p* = 0.049 and 60 μg/kg: *p* < 0.001 versus vehicle). In contrast, the lower doses of ARA 290 did not produce significant relief of allodynia (3 μg/kg: *p* = 0.825 and 10 μg/kg: *p* = 0.707 versus vehicle). Comparing efficacy of treatment with ARA 290, a linear dose response relationship was observed with an adjusted R^2^ of 0.56 (Figure 1B). Higher doses of ARA 290 resulted in higher AUCs corresponding to animals tolerating stimulation with filaments that exert a greater force and hence less mechanical allodynia. Survival analysis indicates that with higher dosages of ARA 290 relief from allodynia persists for longer time periods (Figure 1C, Log-Rank *p* < 0.001).

#### ***Cold allodynia***

**Figure 2:**
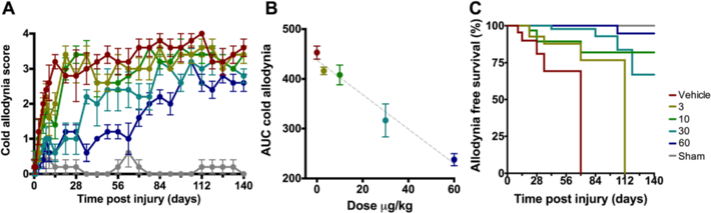
**Effect of ARA 290 on cold allodynia. A**. Effect of spared nerve injury and treatment with vehicle or different doses of ARA 290 on cold allodynia scores. Animals were sham-operated (grey) or received spared nerve injury and 5 doses of vehicle (red), 3 μg/kg ARA 290 (yellow), 10 μg/kg ARA 290 (green), 30 μg/kg ARA 290 (marine blue), or 60 μg/kg ARA 290 (dark blue) administered on days 1, 3, 6, 8 and 10 post-surgery (for each treatment p < 0.0001 compared to vehicle). Scoring of cold allodynia is described in the Methods Section. **B**. Correlation of ARA 290 treatment dose and the relief of cold allodynia, calculated by the difference in area under the curves (AUC) of the cold allodynia score on day 1 vs. day 140. The adjusted R^2^ is 0.78. **C**. Survival analysis showing the cold allodynia-free proportion of animals in time either sham-operated, or receiving spared nerve injury and treated with vehicle or different doses of ARA 290. Log-Rank p < 0.001.

Following SNI, vehicle-treated animals developed cold allodynia within 7–14 days a mean score of 3.2 ± 0.2 (range 3 to 4). Sham operated animals showed no increase in response. Regardless of treatment, all SNI groups differed significantly from sham-operated animals (*p* < 0.001 for all groups). Animals treated with ARA 290 showed a dose-dependent relief of allodynia (Figure 2A, treatment effect *p* < 0.001). *Post hoc* analysis showed that at all doses allodynia was significantly less compared to vehicle (*p* < 0.001). A linear ARA 290 dose–response relationship was observed with an adjusted R^2^ of 0.78 (Figure 2B). Higher doses of ARA 290 resulted in lower AUCs corresponding to animals responding less vigorously to the application of acetone and hence less thermal allodynia. Survival analysis indicates that a more persisting effect was obtained at higher ARA 290 doses (Figure 2C, Log-Rank *p* < 0.001).

### ARA 290 prevents the increase of iba-1-immunoreactivity in the dorsal horn

**Figure 3:**
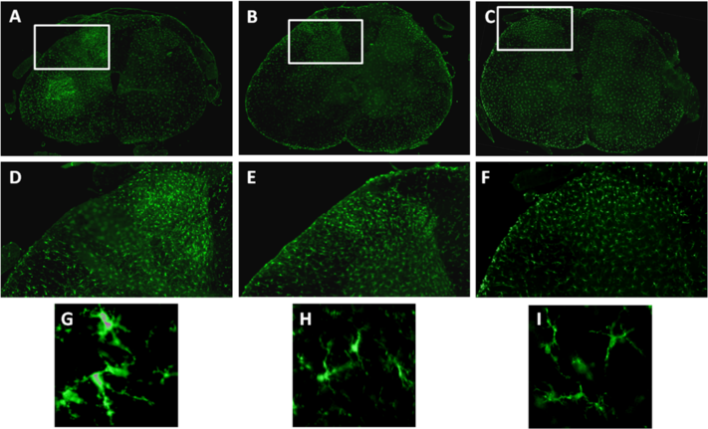
**Effect of ARA 290 on Iba-1 immunoreactivity in the L5 spinal cord segment of animals 2 weeks after spared nerve injury.** Representative photomicrographs of Iba-1 immunoreactivity (green) in the L5 spinal cord segment of animals 2 weeks after spared nerve injury (SNI). **(A-C)** Low power magnifications, **(D-F)** detailed images of **(A-C)** as indicated by the white rectangles, and **(G-I)** high power magnifications of the spinal cord of animals that underwent: **A, D and G**. SNI and vehicle treatment, **B, E and H**. SNI and treatment with 30 μg/kg ARA 290, **C, F and I**. sham surgery without treatment. The left-hand side of the photomicrographs represents the ipsilateral side of the animal, innervating the site of spared nerve injury.

In Figure 3, representative overviews are given from the spinal cords of animals after 2 weeks of survival that received SNI with vehicle (Figure 3A), SNI with 30 μg/kg ARA 290 (Figure 3B) or sham surgery without treatment (Figure 3C). There was an apparent increased iba-1-immunoreactivity (iba-1-IR) on the side of the injury that seemed more pronounced in the 0 μg/kg treated group when compared to the 30 μg/kg group. The dorsal horns of animals that received SNI and treatment with vehicle (Figure 3D), 30 μg/kg ARA 290 (Figure 3E) or sham surgery without treatment (Figure 3F) showed increased iba-1-IR in the dorsal horn which was more pronounced in vehicle-treated animals. High power magnifications of individual microglia from the dorsal horns of animals that received SNI and treatment with vehicle (Figure 3G), 30 μg/kg ARA 290 (Figure 3H) or sham surgery without treatment (Figure 3I). Microglia from the vehicle-treated group showed an activated phenotype with an amoeboid shape and retracted rami, whereas microglia from the 30 μg/kg treatment group and sham surgery group showed a resting phenotype with a stretched soma and rami. For further analysis, computerized calculation of the amount of immunoreactivity was performed.

**Figure 4:**
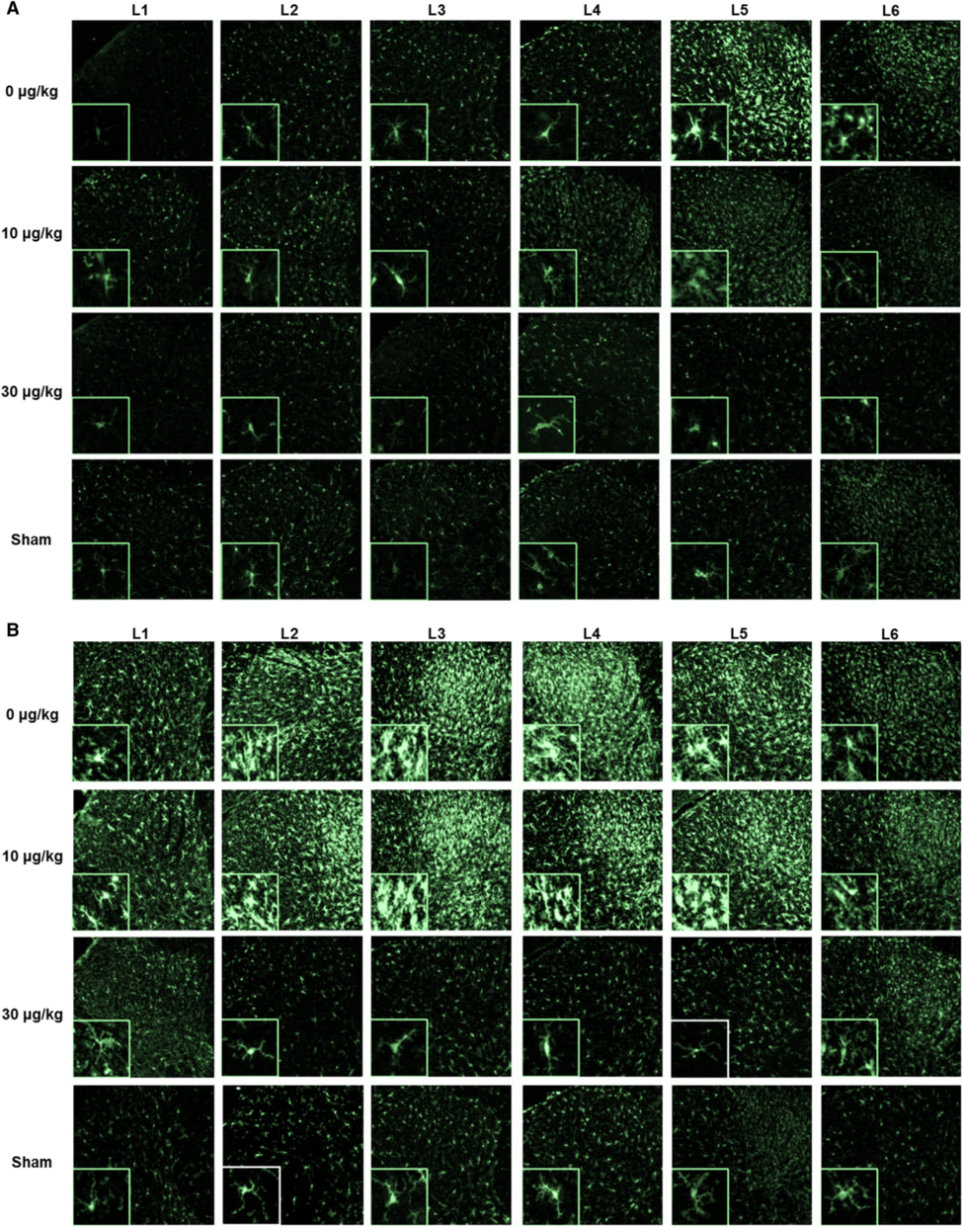
**Effect of ARA 290 on Iba-1 immunoreactivity on L1-L6 spinal cord segments of animals 2 and 20 weeks after spared nerve injury. A**. Representative photomicrographs of Iba-1 immunoreactivity (green) in the lumbar dorsal horns of animals 2 weeks after spared nerve injury (SNI). Animals were either treated with vehicle (upper row), 10 μg/kg ARA 290 (second row) or 30 μg/kg ARA 290 (third row). The bottom row represents sham-operated animals without treatment. In each column, the Iba-1 immunoreactivity signal at different lumbar spinal cord levels (L1-L6) is shown. Inserts show higher magnifications of the photomicrographs. **B**. Representative photomicrographs of Iba-1 immunoreactivity (green) in the lumbar dorsal horns of animals 20 weeks after spared nerve injury (SNI). Animals were either treated with vehicle (upper row), 10 μg/kg ARA 290 (second row) or 30 μg/kg ARA 290 (third row). The bottom row represents sham-operated animals without treatment. In each column, the Iba-1 immunoreactivity signal at different lumbar spinal cord levels (L1-L6) is shown. Inserts show higher magnifications of the photomicrographs.

**Figure 5:**
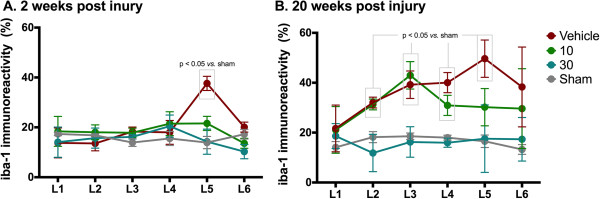
**Effect of ARA 290 on Iba-1 immunoreactivity after spared nerve injury.** Quantification graphs showing Iba-1 immunoreactivity (percentage of immunostained area) in sections of different lumbar spinal cord levels (L1-L6) from animals 2 weeks **(panel A)** or 20 weeks **(panel B)** after sham-operation (grey), spared nerve injury and vehicle-treated (red), spared nerve injury and treatment with 10 μg/kg ARA 290 (green), or spared nerve injury and treatment with 30 μg/kg ARA 290 (marine blue). At 2 weeks post injury **(A)**, Iba-1-IR was increased in the L5 segment following vehicle treatment only (grey box *p* < 0.05 vs. sham). At 20 weeks post injury **(B)**, vehicle *p* < 0.05 vs. sham at segments L2-L5) and 10 μg/kg: *p* < 0.05 vs. sham at segments L2-L4 (grey boxes). Data are mean ± SEM.

Representative images of recorded photomicrographs of iba-1-IR in lumbar dorsal horns L1 to L6 of animals at 2 weeks and 20 weeks following SNI surgery and treated with the various ARA 290 doses are given in Figure 4A and B with high power magnifications of microglia cells presented in the inserts. For group 1 (2 weeks post injury), microglia in the L5 segment of the vehicle treatment group showed an activated phenotype, whereas the microglia in other panels show a resting phenotype. Iba-1-IR was increased in the L5 segment following vehicle treatment only (Figure 5A, *p* < 0.05 versus sham). Irrespective of treatment, no increase in reactivity was observed in any of the other segments. In contrast, in group 2 (20 weeks post injury), iba-1-IR had spread both cranially and caudally to multiple spinal cord segments in vehicle-treated animals (Figure 4B) with significantly increased iba-1-IR in segments L2 to L5 (*p* < 0.05 vs. sham). As shown in the inserts of Figure 4B, microglia in the L1-L6 segments of the vehicle and 10 μg/kg treatment groups showed an activated phenotype, whereas the microglia in the 30 μg/kg and sham groups showed a resting phenotype. In Groups 1 and 2, treatment with 30 μg/kg ARA 290 prevented an increase in iba-1-IR as shown by the absence of iba-1-IR in all segments (Figure 5A and B; 30 μg/kg: ns vs. sham, *p* < 0.05 vs. vehicle at segments L2 to L4). In Group 2, treatment with 10 μg/kg of ARA 290 did not decrease iba-1-IR relative to vehicle-treated animals (Figure 5B; ns vs. vehicle, *p* < 0.05 vs. 30 μg/kg at segments L2-L4).

### SNI does not increase GFAP-immunoreactivity in the dorsal horn

**Figure 6:**
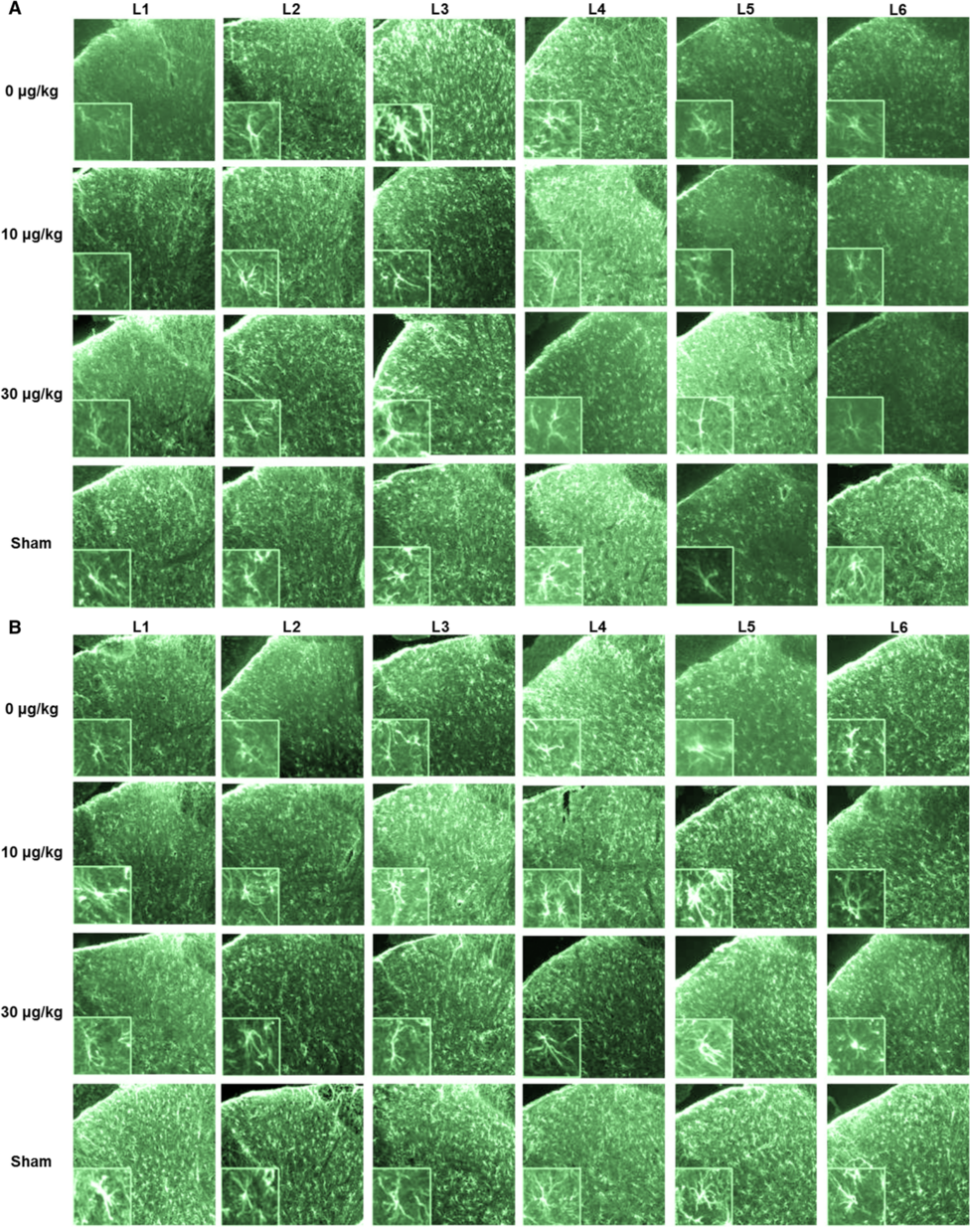
**Effect of ARA 290 on GFAP immunoreactivity on L1-L6 spinal cord segments of animals 2 and 20 weeks after spared nerve injury. A**. Representative photomicrographs of GFAP immunoreactivity (green) in the lumbar dorsal horns of animals 2 weeks after spared nerve injury (SNI). Animals were either treated with vehicle (upper row), 10 μg/kg ARA 290 (second row) or 30 μg/kg ARA 290 (third row). The bottom row represents sham-operated animals without treatment. In each column, the GFAP immunoreactivity signal at different lumbar spinal cord levels (L1-L6) is shown. Inserts show higher magnifications of the photomicrographs. **B**. Representative photomicrographs of GFAP immunoreactivity (green) in the lumbar dorsal horns of animals 20 weeks after spared nerve injury (SNI). Animals were either treated with vehicle (upper row), 10 μg/kg ARA 290 (second row) or 30 μg/kg ARA 290 (third row). The bottom row represents sham-operated animals without treatment. In each column, the GFAP immunoreactivity signal at different lumbar spinal cord levels (L1-L6) is shown. Inserts show higher magnifications of the photomicrographs.

**Figure 7:**
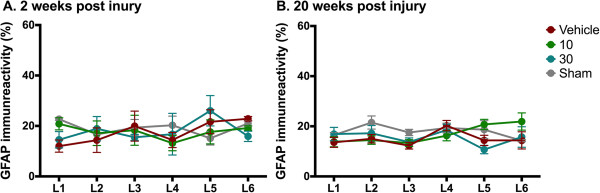
**Effect of ARA 290 on GFAP immunoreactivity after spared nerve injury.** Quantification graphs showing GFAP immunoreactivity (percentage of immunostained area) in sections of different lumbar spinal cord levels (L1-L6) from animals 2 weeks **(panel A)** or 20 weeks **(panel B)** after sham-operation (grey), spared nerve injury and vehicle-treated (red), spared nerve injury and treatment with 10 μg/kg ARA 290 (green), or spared nerve injury and treatment with 30 μg/kg ARA 290 (marine). Data are mean ± SEM.

SNI did not induce an astrocytic response in vehicle-treated animals relative to sham-operated rats for spinal cord segments L1-L6 at either 2 weeks or 20 weeks post injury (Figures 6 and 7). No effect of treatment was observed.

## Discussion

The main findings of this study are: (1) The spared nerve injury model caused a rapid and long-lasting neuropathy with mechanical and cold allodynia; (2) ARA 290 produced dose-dependent relief of both mechanical and cold allodynia; (3) A spreading microglia response (i.e. iba-1-IR and phenotype) was apparent from L5 at week 2 following nerve damage to L2-6 at week 20; (4) No effect of nerve injury on the astrocyte response was observed at weeks 2 and 20 following nerve damage; and (5) ARA 290 suppressed iba-1R in a dose dependent manner.

Neuropathic pain in animals (due to experimental nerve damage) and humans (due to sarcoidosis or diabetes mellitus type 2) responds well to treatment with ARA 290, in that it produces relief of spontaneous pain (humans) and allodynia (humans and animals) [25,26,31,35]. Studies in mice that lack the β-common-receptor show further that ARA 290 is without behavioral effect (ie*.* allodynia is not relieved by ARA 290), implicating this receptor as site of action of ARA 290 [25,26]. The β-common-receptor forms a heterocomplex together with EPO receptor and it is believed that this receptor complex, which we designate the innate repair receptor (IRR), is the molecular site of action of both EPO and ARA 290 [27,29,36]. Exogenous EPO, similar to ARA 290, reverses allodynia and reduces neuronal apoptosis and proinflammatory cytokine production, neuronal regeneration and the release of anti-inflammatory cytokines [28]. We do not use EPO in our studies as, in contrast to ARA 290, it comes with severe side effects including enhanced hematopoiesis and cardiovascular complications (eg*.* hypertension, thrombosis, myocardial infarction). In common with previous studies [25,26], we show here that ARA 290 has effective and prolonged (up to 20 weeks) anti-allodynic effects.

There is ample evidence that peripheral nerve injury results in a strong spinal inflammatory response [17]. For example, we previously showed in mice that surgical damage to the sciatic nerve causes the increase of expression of pro-inflammatory markers including iba-1 mRNA, GFAP mRNA and CCL2 mRNA, within 7 days following nerve damage [26]. CCL2 plays an important role in the invasion of monocytes from peripheral blood as well as resident macrophages towards the spinal cord lesion site following peripheral nerve damage. In our current study the inflammatory response following SNI was apparent from the increase in iba-1-IR. The iba-1-IR response showed a marked expansion from level L5 in week 2 following SNI, to 5 adjoining segments, L2 to L6, at week 20. In addition to the spreading of iba-1-IR to multiple segments, the intensity of the response also increased over time as shown by a higher degree of iba-1-IR and phenotypic signs of activation. We are the first to show this spreading inflammatory response in the spared nerve injury model of neuropathic pain. Similar observations were made earlier in experimental models of spinal cord injury and nerve root avulsion [37,38]. Previous reports of glial response following peripheral nerve injury showed that the response area is confined to the spinal cord segments innervated by the damaged nerve [39,40]. However, these responses were measured within a 2-week time frame. This is in agreement with our observation of lack of spreading at week 2. Caudal and cranial expanding inflammation, as observed here, may explain the increase in severity of NP symptoms over time and development of symptoms in areas of the body not innervated by the damaged nerve [41]. Similarly, various experimental reports indicate that the inflammatory responses may spread to contralateral spinal cord areas [8,42]. In this study we did not quantify contralateral inflammation. Our data do suggest that time is an important factor in the spreading of the microglia response.

Iba-1-IR reflects microglia activation in addition to localization and morphology. Our data show increased iba-1-IR after SNI, which is dose-dependently and long-term reduced by ARA 290 treatment coupled to a dose-dependent and long-term reduction of mechanical and cold allodynia. This long-term effect suggests a disease modulatory effect of ARA 290. We argue that ARA 290 initiates a cascade of events involving several transduction factors of which activation of the IRR is the first step that eventually silences or reduces the inflammatory process [29,36]. Since the activation or recruitment of microglia is largely mediated through the local production of CCL2 [43,44], a possible scenario is that ARA 290 reduces the release of CCL2 via activation of the IRR on neuronal and immune cells [26]. However, both at 2 and 20 weeks after SNI and ARA 290 treatment, relief of allodynia was not complete, indicating that the central response to peripheral nerve damage involves multiple systems including neuroinflammation and probably also up-regulation of excitatory pathways and synaptic plastic changes. Of interest is that ARA 290 treatment causes a reduction in NMDA mRNA (subunits NR1, NR2A and NR2B) in SNI animals, suggestive of an additional role, apart from immune-modulation, for ARA 290 in the treatment of neuropathic pain by suppression of excitatory glutamatergic activity [26].

In contrast to a markedly increased iba-1-IR after SNI, no change in GFAP-IR or astrocytic phenotype (ie*.* activation) was observed in animals with an SNI treated with vehicle after 2 and 20 weeks of lesion. This observation stands in contrast with reports describing involvement of astrocytes adjacent to microglia in NP [5,7,10,45,46]. The absence of astrocytosis in our SNI model may be explained by a time-limited astrocyte response (ie*.* < 2 weeks or between 2–20 weeks). The involvement of astrocytes following peripheral nerve injury reported in the literature varies with some studies showing a relatively short-lived increase in GFAP-IR [7], while others show an increase in GFAP-IR after 14 days that was still present after 150 days [5]. Further studies using more dense observations over time are required to get a reliable indication of the kinetics of the astrocyte response to peripheral nerve damage.

We have argued that the spinal cord is the predominant site of action of ARA 290 following peripheral nerve damage. Indeed, there is ample evidence that peripheral nerve injury activates an innate immune response activated in the spinal cord [5-11]. Furthermore, a complete block of the peripheral nerve with local anesthetics will not prevent central inflammation following peripheral nerve damage but only delays the development of pain, suggestive of a predominant central effect [47,48]. Still, at this point we cannot exclude an additional peripheral effect of ARA 290. Indeed, EPO specifically reduces axonal TNF-α in Schwann cells after peripheral nerve injury, resulting in attenuation of NP symptoms [49]. Hence, in addition to a central nervous system effect, modulation of the peripheral nerve immune response could also be part of the mechanism of action of ARA 290, but these specific effects remain to be investigated.

## Conclusions

In conclusion, in the spared nerve injury model, we show that the erythropoietin-analogue ARA 290 dose-dependently reduces allodynia coupled to suppression of the spinal microglia response, suggestive of a mechanistic link between ARA 290-induced suppression of central inflammation and relief of neuropathic pain symptoms.

## Methods

### Animals

The experimental protocol was approved by the Animal Ethics Committee (Dierethische Commissie) of the Leiden University Medical Center, Leiden, The Netherlands and the Animal Care and Use Review Office (ACURO) of the United States Army Medical Department Medical Research and Materiel Command. All experiments were performed in accordance to the guide lines of the International Association for the Study of Pain [50]. Forty-two, eight-week-old, female Sprague–Dawley rats (Charles River, Maastricht, The Netherlands) weighing 200 to 260 grams were used in this study. Animals were housed two per cage in individually ventilated cages for the duration of the entire experimental period under standard laboratory conditions with water and food *ad libitum* and a 12 h-12 h light/dark cycle. At the end of the studies the animals were anesthetized and euthanized by exsanguination under 6% sevoflurane anesthesia, perfuse-fixed with 100 ml ice-cold heparinized saline followed by 150 ml 4% paraformaldehyde for tissue extraction.

### Neuropathic pain model

Chronic neuropathic pain was induced in 34 rats by spared nerve injury (SNI) [25]. Animals were anesthetized with 6% sevoflurane induction and 3% maintenance. A small incision was made in the lateral surface of the left thigh of the animal, exposing the muscles. The trifurcation of the sciatic nerve was revealed by blunt preparation between the two heads of the biceps femoris muscle. Next, the tibial and common peroneal nerves were tightly ligated with 5–0 silk in rats and cut to remove 2–4 mm of the distal nerve. The sural nerve was left intact. In order to prevent spontaneous nerve reconnection, the transected nerves were displaced. During the surgical procedure, great care was taken not to stretch or touch the sciatic or sural nerves. The wound was closed in one layer with 4–0 ethilon and a single dose of 0.01 mg/kg buprenorphine was administered to relieve acute postoperative pain.

Eight animals received a sham operation where the nerve was exposed, but not ligated and transected. The wound was closed in one layer with 4–0 ethilon sutures and a single dose of 0.01 mg/kg buprenorphine was administered for the relief of acute postoperative pain. After surgery, animals were allowed to recover with body temperature maintained at 38°C for 1 h before being transferred to a cage with fresh saw dust.

### Treatment

The experimental drug ARA 290 was dissolved in phosphate buffered saline (PBS) to obtain a stock solution, aliquoted and stored at 4°C until use. Prior to injection, the stock solution of was diluted in PBS to yield the desired dose in 200 μl. Following surgery, 34 animals were treated on days 1, 3, 6, 8 and 10 post-surgery. All injections were administered intraperitoneally. Nine of the animals were sacrificed after the 2-week treatment period (Group 1); twenty-five animals were followed for another 18-weeks following treatment and then sacrificed (Group 2). Group 1 animals were randomly allocated to one of the following treatment groups: 0 (= vehicle; PBS), 10, 30 μg/kg (*n* = 3/group). Group 2 animals were randomly allocated to one of the following treatment groups: 0 (= vehicle; PBS), 3, 10, 30, 60 μg/kg ARA 290 (*n* = 5/group). Three animals in Group 1 and five in Group 2 received sham surgery and were not treated (*ie.* sham controls).

### Neuropathic pain assay

Allodynia was assessed prior to surgery (baseline values), at days 1, 3, 6, 8 and 10 during the treatment period, and during follow up from day 14 on at 1-week intervals. The test site was the plantar surface of the injured hind paw. The animals were placed in a transparent cage on an elevated wire mesh floor and were allowed to habituate for at least 10 minutes before testing for mechanical allodynia, followed by thermal allodynia after a short interval to allow recovery from the previous test. When testing coincided with a treatment day, testing was performed prior to administration of ARA290 or vehicle.

Mechanical allodynia was tested with the use of von Frey hairs (Semmes-Weinstein Monofilaments, North Coast Medical Inc., San Jose, CA) with increasing stiffness (0.004 – 300 g) causing incremental forces to be exerted on the plantar surface of the injured hind paw. The hairs were applied 10 times at a frequency of 1 Hz to slightly different loci within the test area to avoid sensitization due to repetition. The force necessary to evoke a pain reflex by a brisk paw withdrawal was recorded and no further filaments were applied to the paw that showed a response. All measurements were obtained in duplex with a 1-minute interval between the tests and then averaged.

Cold allodynia was tested by using the acetone test. Fifty microliters of acetone was sprayed on the plantar surface of the hind paw. The response of the animal was recorded using the following classification: 0 = no withdrawal, 1 = startle response lasting less than 1 s, 2 = withdrawal lasting between 1 and 5 s, 3 = withdrawal lasting between 5 and 30 s (with or without paw licking) and 4 = withdrawal lasting longer than 30 s (with or without licking and repeated shaking). All measurements were obtained in duplex with a 1-min interval between the tests and then averaged.

### Immunohistochemistry

After perfuse-fixing the animals, the lumbar spinal cord was extracted and post-fixed in 4% paraformaldehyde for 24 h. After post-fixation, the tissues were cryoprotected for 72 h in 30% sucrose before embedding them in TissueTek (Sakura FineTek, Alphen a/d Rijn, The Netherlands). The extracted lumbar spinal cord was sectioned transversally at a freezing microtome at -20°C to obtain serially sectioned 20 μm sections. Every 10th section was mounted on a Superfrost + slide (Menzel Gläser, Braunschweig, Germany) and stored at -80°C prior to staining. For immunohistological staining, sections for all animals of both time points were stained in one run for each antibody to reduce variability between stainings. The sections were retrieved from the freezer and allowed to thaw before blocking for 1 h with 10% goat serum (Invitrogen, Auckland, New Zealand) with 0.4% Triton X-100 (Sigma-Aldrich, St. Louis, USA). Sections were stained overnight at 4°C for microglia with 1 μg/ml rabbit-anti-Iba-1 (Wako Chemicals GmbH, Neuss, Germany) or astrocytes with 1:200 rabbit-anti-GFAP (Dako, Heverlee, Belgium) in 3% normal goat serum with 0.4% Triton X-100. After 3 washings in PBS, the slides were incubated for 3 h at room temperature with 1:500 goat-anti-rabbit-Alexa488 (Invitrogen, Eugene, USA) as a secondary antibody in 3% normal goat serum with 0.4% Triton X-100. After incubation, slides were washed 3 times with PBS and Vectashield (Vector Laboratories Inc., Burlingame, USA) as an anti-fading agent was applied. Lastly, the slides were cover slipped and sealed with nail polish. Standardized microphotographs of the dorsal horn were taken with a Leica M5500 fluorescence microscope (Leica Microsystems, Rijswijk, The Netherlands). During photography, the spinal cord segment of the image was determined with a spinal cord histology atlas on the basis of white matter to grey matter ratio, ventral horn morphology and dorsal horn morphology and documented for classification during analysis [51]. The photomicrographs were analyzed using ImageJ (NIH, Bethesda, MD, USA).

### Image analysis

First, images were screened for quality by assessing if the dorsal horn was completely visible, without folds or significant damage. Images that did not meet these criteria were not analyzed (on average 5.3% per group). Next, the remaining 8-bit grey scale images were thresholded using the auto threshold function of ImageJ to create dichromatic images required for analysis of the percentage covered with immunoreactive cells. This function objectively separates signal from noise and no adjustments for background, brightness or contrast were performed. Obtained values were averaged per spinal cord segment for each animal; the images given represent these average responses.

### Statistics

#### ***Allodynia***

Behavioral data for effects on tactile and cold allodynia were analyzed by 2-way analysis of variance with *post hoc* Student-Newman-Keuls comparisons for multiple testing. The effect of dose-dependency was analyzed by calculating the area under the curve with the trapezoid rule and curve fitting the data using a linear function. Log-Rank survival curves were created to determine the duration of allodynia relief by ARA 290 treatment. End-points were defined as reaching the maximum amount of measurable allodynia (reaching the 0.004 g filament or reaching a score of 4 in the acetone test). Holm-Sidak *post hoc* analysis for multiple comparisons was performed.

#### ***Microscopy***

Spinal cord microscopy data were analyzed per segment by two-way analysis of variance with *post hoc* Student-Newman-Keuls comparisons for multiple testing.

All data are presented as mean ± SEM unless otherwise stated. *p-*values < 0.05 were considered significant.

## Competing interests

Ann Dunne, Michael Brines and Anthony Cerami are employees of Araim Pharmaceuticals Inc. (Tarrytown, NY, USA), which is developing nonerythropoietic tissue protective compounds for clinical use. This does not alter the authors’ adherence to all the Molecular Pain policies on sharing data and materials. The other authors declare no conflict of interest or competing interests.

## Authors’ contributions

Conceived and designed the experiments: A. Dahan, MS, AC and MB. Performed the experiments: A. Dahan, MS, MN, CCH and HSK. Analyzed the data: A. Dahan, MS, MN, CCH and MvV. Contributed reagents/materials/analysis tools: A. Dahan, MS and CCH. Wrote the paper: A. Dahan, MB, MS, AC, LA, A. Dunne and MvV. All authors read and approved the final manuscript.

## Support

This work was supported by grant 101498960 from the United States Department of Defense Medical Research and Development Program, Washington, D.C.
